# Evaluating a couple-based intervention addressing sexual concerns for breast cancer survivors: study protocol for a randomized controlled trial

**DOI:** 10.1186/s13063-019-3975-2

**Published:** 2020-02-12

**Authors:** Jennifer Barsky Reese, Lauren A. Zimmaro, Stephen J. Lepore, Kristen A. Sorice, Elizabeth Handorf, Mary B. Daly, Leslie R. Schover, Deborah Kashy, Kelly Westbrook, Laura S. Porter

**Affiliations:** 1 Cancer Prevention and Control Program, Fox Chase Cancer Center, 333 Cottman Avenue, Philadelphia, PA 19111 USA; 2 Department of Social and Behavioral Sciences, College of Public Health, Temple University, 1301 Cecil B. Moore Avenue, Ritter Annex, Philadelphia, PA 19122 USA; 3 Department of Biostatistics and Bioinformatics, Fox Chase Cancer Center, 333 Cottman Avenue, Philadelphia, PA 19111 USA; 4 Department of Clinical Genetics, Fox Chase Cancer Center, 333 Cottman Avenue, Philadelphia, PA 19111 USA; 5 Will2Love LLC, 1333 Old Spanish Trail, Suite G, #134, Houston, TX 77054 USA; 6 Department of Psychology, Michigan State University, 316 Physics Road, Room 262, East Lansing, MI 48824 USA; 7 Department of Medicine-Oncology, Duke University Medical Center, DUMC 3459, Durham, NC 27710 USA; 8 Department of Psychiatry & Behavioral Sciences, Duke University Medical Center, DUMC 90399, Durham, NC 27708 USA

**Keywords:** Breast cancer, Sexual dysfunction, Intimacy, Telephone-based, Randomized controlled trial

## Abstract

**Background:**

Sexual concerns are distressing for breast cancer survivors and interfere with their intimate relationships. This study evaluates the efficacy of a four-session couple-based intervention delivered via telephone, called Intimacy Enhancement (IE). The IE intervention is grounded in social cognitive theory and integrates evidence-based techniques from cognitive behavioral couple therapy and sex therapy to address survivors’ sexual concerns and enhance their and their partners’ sexual, relationship, and psychological outcomes.

**Methods:**

This trial is designed to evaluate the efficacy of the IE intervention in improving survivors’ sexual function, the primary study outcome. Secondary outcomes include survivors’ sexual distress, partners’ sexual function, and survivors’ and partners’ relationship intimacy and quality as well as psychological distress (depressive symptoms and anxiety symptoms). Additional aims are to examine whether treatment effects on patient sexual function are mediated by sexual communication and self-efficacy for coping with sexual concerns and to explore whether survivor age and race/ethnicity moderate intervention effects on survivors’ sexual function. Eligible adult female breast cancer survivors reporting sexual concerns and their intimate partners are recruited from two academic sites in the USA and are randomized to either the IE intervention or to a control condition of equal length offering education and support around breast cancer-related health topics (Living Healthy Together). The target sample size is 120 couples. Self-report outcome measures are administered to participants in both conditions at baseline (T1), post-treatment (T2), 3 months post-treatment (T3), and 6 months post-treatment (T4).

**Discussion:**

Evidence-based interventions are needed to address sexual concerns for breast cancer survivors and to enhance their and their intimate partners’ sexual, relationship, and psychological well-being. This randomized controlled trial will allow us to examine the efficacy of a novel couple-based intervention delivered via telephone for breast cancer survivors experiencing sexual concerns and their intimate partners, in comparison with an attention control. Findings of this study could influence clinical care for women with breast cancer and inform theory guiding cancer-related sexual rehabilitation.

**Trial registration:**

ClinicalTrials.gov, NCT03930797. Registered on 24 April 2019.

## Background

Nearly one-third of all new cancer diagnoses in women are for breast cancer [1]. The majority of women are diagnosed with localized breast cancer, for which the 5-year survival rate is 99% [1]. Advances in detection and treatment have improved survival for breast cancer, yet these life-extending treatments can come at a considerable cost for survivors’ intimate relationships [2, 3], which are often the cornerstone of their support systems. As many as 70% of breast cancer survivors report sexual concerns related to cancer diagnosis or treatment [4, 5]. Common concerns include those that are biological (e.g., vaginal dryness, pain during sex) [6–8], psychological (e.g., loss of sexual desire) [9, 10], or social in nature (e.g., changes in partnered sexual activity) [11–14]. Some of the most chronic and distressing sexual concerns for breast cancer survivors result from the estrogen suppression effects of chemotherapy, hormone therapy (e.g., aromatase inhibitors), and ovarian suppression [6, 15–17]. In addition, post-surgery body changes, including the loss of breast and nipple sensitivity, can interfere with sexual activity and impede women’s sexual arousal, a key component of their sexual function [8, 9, 18, 19]. Loss of sexual desire is among the most problematic issues because it can significantly disrupt women’s intimate relationships [20]. In turn, relationship factors can be strong predictors of sexual function [7, 12, 21, 22].

In contrast with many areas of health-related quality of life (QOL) that tend to improve over time, sexual concerns often persist for years after breast cancer survivors complete their primary treatments [5, 23–25]. As a result, many breast cancer survivors and their partners may wish to resume a satisfying intimate relationship after treatment ends but encounter difficulties in doing so. If unaddressed, sexual concerns can lead to clinically significant psychological distress [5, 20] and may compromise survivors’ relationships and quality of life [2, 17, 26]. By contrast, there is evidence that addressing sexual concerns can have positive benefits not only for the survivors’ sexual outcomes but also for other aspects of their and their partners’ individual and relationship well-being [27–30]. In light of such findings, reviews of interventions in this area have concluded that the most effective interventions for addressing breast cancer survivors’ sexual concerns tend to be those that are couple-based [27, 31]. To summarize, sexual concerns detract from the well-being of breast cancer survivors, whereas addressing such concerns might assist with preserving survivors’ well-being while also benefiting their partners.

A couple-based intervention that systematically involves the partner may be a highly effective tool for addressing survivors’ sexual concerns and enhancing their sexual function for several reasons [31–34]. First, breast cancer-related sexual concerns are most often experienced in the context of partnered sexual activity [15, 23]. Therefore, survivors may benefit from guidance in putting physical aids (e.g., vaginal lubricants) and behavioral skills (e.g., communication skills) into practice in their intimate relationships [35–39]. Second, partners of breast cancer survivors commonly report sexual function problems [40] as well as difficulties in adjusting to their partners’ sexual and body changes [11]. For instance, they may avoid touching their partner’s breast or chest area or sexual activity altogether out of fear that they may hurt their partner physically or burden her with sexual demands. If not addressed, such factors could compromise survivors’ efforts at coping effectively with sexual problems. Third, breast cancer survivors are most likely to discuss their sexual concerns with their intimate partners compared to other social outlets [41] and report a preference to have their partners involved in sexuality interventions [20]. Despite these factors, few studies of sexuality interventions in breast cancer have targeted couples by systematically including partners [42, 43].

Therefore, this study aims to evaluate a couple-based intervention designed to address sexual concerns for breast cancer survivors. The intervention, called Intimacy Enhancement (IE), is designed to be delivered via telephone to reduce participant burden and increase accessibility. The IE intervention is grounded in social cognitive theory [44] which posits that self-efficacy, i.e., the confidence that one can successfully complete a behavior, is a key predictor of accomplishing that behavior [45, 46]. Self-efficacy has been shown to be a critical process underlying successful behavior change interventions in cancer [47, 48] that is best targeted through skills practice [46]. The IE intervention integrates skills practice and applies effective practices drawn from cognitive behavioral couple therapy (e.g., communication skills training) [49, 50] and sex therapy (e.g., sensate focus) [51, 52]. This intervention was adapted from a similar intervention initially developed for use with colorectal cancer survivors and their partners after finding promising effects on a range of patient and partner sexual and relationship outcomes [53, 54]. The adaptation was informed by a qualitative research study with breast cancer survivors to optimize the relevance of the educational content and of the skills training and practice for this new population [20].

We initially conducted a randomized pilot trial of the IE intervention in a sample of breast cancer survivors and their intimate partners and found support for the intervention’s feasibility and acceptability as well as promising effects on key sexual and psychosocial outcomes [55]. We thus planned the current full-scale trial to evaluate the IE intervention’s efficacy, investigate mediators of treatment effects, and explore potential intervention moderators. This trial compares the IE intervention with a previously tested attention control condition providing breast cancer-related education and support called Living Healthy Together (LHT). The IE intervention skills training activities are designed to increase patients’ self-efficacy for coping with sexual concerns and improve their ability to communicate with their partners about sex; our pilot work found evidence supporting these treatment effects [55]. We therefore expect that increased self-efficacy for coping with sexual concerns and improvements in sexual communication will be mechanisms through which the IE intervention improves patient sexual function, and will test this in mediation analyses. The IE intervention appears to be acceptable and relevant across a diverse sample of patients of various ages and racial/ethnic groups. However, it is possible that the age and race/ethnicity of participants could influence sexual relationships [22, 56–60] and thus might interact with the IE intervention. We will therefore examine this in exploratory moderator analyses. This manuscript describes the study protocol for a multisite randomized controlled trial evaluating the IE intervention, a couple-based intervention addressing sexual concerns for post-treatment breast cancer survivors reporting sexual concerns.

### Study aims

**Fig. 1:**
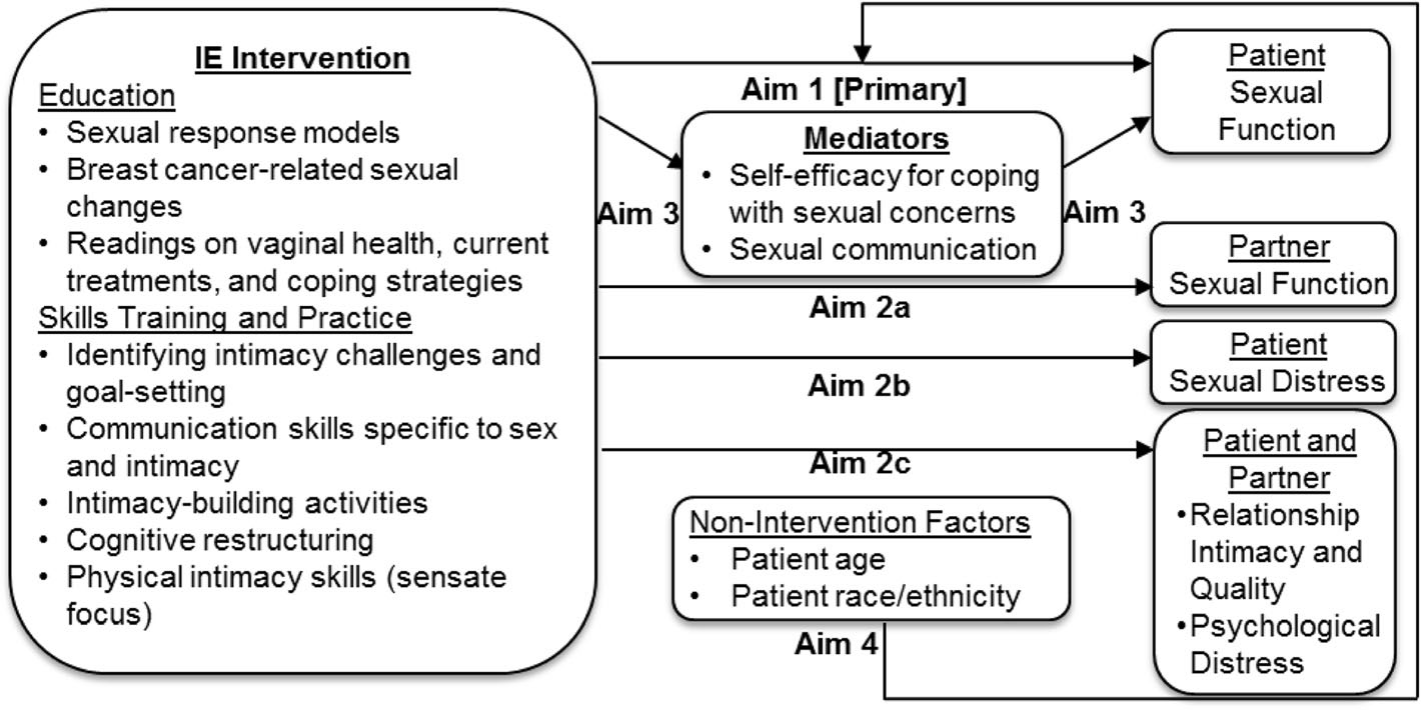
Study conceptual schematic

The specific aims of this study are illustrated in Fig. 1 and are as follows:

1. To evaluate whether the IE intervention leads to a significantly greater increase in patient sexual function from pre-treatment to post-treatment and at 3 and 6 month follow-ups compared to the LHT condition (Aim 1; Primary).
2. To evaluate whether the IE intervention leads to significantly greater improvements in partner sexual function (Aim 2a; Secondary), patient sexual distress (Aim 2b; Secondary), patient/partner relationship intimacy/quality, and psychological distress (Aim 2c; Secondary) from pre-treatment to post-treatment and at 3 and 6 month follow-ups, compared to the LHT condition.
3. To evaluate whether increases from pre- to post-treatment in patient sexual communication and self-efficacy for coping with sexual concerns mediate the beneficial effects of the IE intervention on patient sexual function at the 3 and 6 month follow-ups (Aim 3; Secondary).
4. To explore whether age (< 45 versus > 45 at diagnosis) and race/ethnicity (White versus non-White) moderate intervention effects on the primary outcome of patient sexual function (Aim 4; Exploratory).

## Methods/design

### Study design

**Fig. 2:**
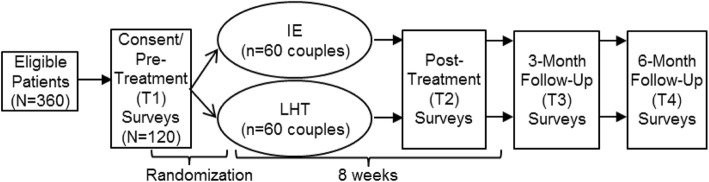
Project flow diagram

A two-group randomized controlled trial design with pre-test and repeated post-test measures is used to accomplish the study aims. Women with breast cancer (*N* = 120) and their partners are randomized to one of two intervention conditions with equal allocation: Intimacy Enhancement (IE) or Living Healthy Together (LHT). Randomization is stratified by age at diagnosis and recruitment site. Both interventions are manualized, of equivalent duration, and delivered by a trained counselor to the couples jointly over the telephone. Web-based self-report outcome measures are administered to participants at baseline (T1), post-treatment (T2), 3 months post-treatment (T3), and 6 months post-treatment (T4). The study design is guided by the Consolidated Standards of Reporting Trials (CONSORT) criteria [61], and the project flow is shown in Fig. 2. The Standard Protocol Items: Recommendations for Interventional Trials (SPIRIT) guidelines [62] have been followed for this protocol, and the schedule of enrollment, interventions, and assessments is shown in Table 1. The SPIRIT checklist is provided as an additional file (see Additional file 1). All items from the current registry can be found within this protocol. Patient recruitment and data collection began in May 2019.

**Table 1:** Schedule of enrollment, interventions, and assessments

	Enrollment	Allocation	Post-allocation			
	Eligibility T0	Baseline T1	Sessions 1–4	Post-Session T2	3 Months T3	6 Months T4
ENROLLMENT						
Eligibility screen	X					
Informed consent		X				
Allocation		X				
INTERVENTIONS						
IE intervention			X			
LHT intervention			X			
MEASURES						
Primary outcome						
Female Sexual Function Index (FSFI)^a^		X		X	X	X
Secondary outcomes						
Male partners: International Index of Erectile Function (IIEF) Female partners: FSFI		X		X	X	X
Female Sexual Distress Scale- Revised (FSDS-R)^a^		X		X	X	X
Miller Social Intimacy Scale (MSIS)		X		X	X	X
Dyadic Adjustment Scale-7 item (DAS-7)		X		X	X	X
Patient Health Questionnaire-9 item (PHQ-9)		X		X	X	X
Generalized Anxiety Disorder 7-item (GAD-7)		X		X	X	X
Mediators						
Dyadic Sexual Communication Scale (DSCS)^a^		X		X		
Self-efficacy (for coping with sexual concerns)^a^		X		X		

^a^A patient-only measure

### Participants

Eligibility inclusion criteria are as follows: female adults who have a medically confirmed diagnosis of localized breast cancer (stages T1–T4, N0–N1, and M0); age > 18 years; completed active treatment (surgery, chemotherapy, immunotherapy/targeted therapy, radiation therapy) 6 months to 5 years prior (current endocrine therapy use is acceptable); live with a partner (same or opposite sex) > 6 months and in a relationship that could involve sexual activity; partner or spouse age > 18 years; and report sexual concerns, as determined by scores > 3/10 on an item adapted from the Patient Care Monitor (PCM) [63], a reliable indicator of sexual concerns for women with breast cancer [2]. Exclusion criteria are the following: patient has a past or current history of cancer other than non-melanoma skin cancer including a prior breast cancer; patient or partner cannot speak and read in English; patient and partner do not have reliable telephone access; patient or partner has a hearing impairment that precludes participating in a telephone intervention; patient or partner has an Eastern Cooperative Oncology Group (ECOG) [64] score > 2 or is deemed medically unable to participate; patient has overt cognitive dysfunction or psychiatric disturbance; couple is currently in marital/couple therapy; patient is currently pregnant; and partner does not agree to participate.

### Procedures

#### Screening and consent

Patients are recruited through Fox Chase Cancer Center (FCCC) and Duke University Medical Center (DUMC), National Cancer Institute (NCI)-designated comprehensive cancer centers in the USA located in urban or suburban locations. Potentially eligible candidates are identified from each provider’s clinic schedules, institution tumor registries, or clinician referral. Introductory study letters are sent to preliminarily eligible patients. Patients who do not decline further contact are contacted by a member of the study team who provides information about the study, screens the patient for eligibility, and reviews study procedures with the couple. Study advertisements supplement mailing-based recruitment. Recruiting couples is quite challenging [65, 66]. If necessary, we will also expand recruitment efforts by working with community partners who provide support services to patients with cancer.

Consent is obtained using web-based forms, although participants are given the option to complete paper-based consent forms. Consent is considered complete when both members of the couple consent. Patients and partners receive a total possible compensation for the study of $140 per person or $280 per couple in either gift card or debit card form. Retention in our pilot trial was excellent [55], and we are employing similar methods to reduce participant burden and enhance retention in this trial, including web/mail-based surveys, telephone sessions, and flexible scheduling of sessions.

#### Data collection

Data collection is completed using Research Electronic Data Capture (REDCap), a secure, Health Insurance Portability and Accountability Act (HIPAA)-compliant web-based application used routinely in randomized controlled trials. Data collection using REDCap could reduce error due to manual data entry. Forms are routinely checked for quality. Participants without computer access can complete measures using paper and pencil versions. We have selected 3 and 6 month follow-ups to facilitate the evaluation of maintenance of IE intervention effects over a 6-month period, during which sexual concerns would otherwise likely be stable [2]. Various methods are employed to keep participant data confidential and secure including using password-protected files, limiting access to only those on the study team who require identifiable data, and using de-identified data when possible.

#### Randomization

This study uses 1:1 blocked randomization (group size 4), stratifying by age at diagnosis (< 45 versus > 45) [9, 56] and recruitment site. The project biostatistician generated the randomization sequence, and treatment assignment occurs through REDCap. Randomization occurs once the couple has completed their baseline assessment and is scheduled for their first intervention session. Couples receive sealed study materials and are instructed to leave the envelopes sealed until Session 1 (to minimize unequal drop-out after participants know their allocated study arm). As with most behavioral interventions, study interventionists, intervention supervisors (site principal investigators [PIs]), and participants are not blind to study condition. To facilitate unbiased data collection and analysis, the following steps are taken: (1) the study biostatistician will conduct outcome data analyses on data in which the study condition is masked; (2) baseline (T1) surveys are administered prior to randomization; and (3) outcome data collection is completed in an automated fashion using REDCap, minimizing the need for contact with participants to collect study data. During the annual review of adverse events, the presence of significant between-group differences could potentially warrant unmasking of the study conditions by the study biostatistician.

### Measures

#### Overview of measures

The measures used in this trial have been shown to be reliable and valid when used in studies of patients with cancer. In addition, to reduce the burden of survey completion for study participants, brief surveys have been selected and abbreviated or short-form versions of measures are selected when available. Full descriptions of the outcome measures (i.e., outcome definitions) are shown in Table 2.

**Table 2:** Outcome definitions

Domain	Measure	Metric	Method of aggregation	Time points (for comparison)
	Primary outcome			
Sexual function	Female Sexual Function Index (FSFI)^a^	Difference in change scores	Mean score	T1–T2; T1–T3; T1–T4
	Secondary outcomes			
Sexual function	Male partners: International Index of Erectile Function (IIEF) Female partners: FSFI	Difference in change scores; difference in proportions	Mean score; proportions by clinical cut-off score	T1–T2; T1–T3; T1–T4
Sexual distress	Female Sexual Distress Scale- Revised (FSDS-R)^a^	Difference in change scores	Mean score	T1–T2; T1–T3; T1–T4
Relationship intimacy	Miller Social Intimacy Scale (MSIS)	Difference in change scores	Mean score	T1–T2; T1–T3; T1–T4
Relationship quality	Dyadic Adjustment Scale-7 item (DAS-7)	Difference in change scores	Mean score	T1–T2; T1–T3; T1–T4
Psychological distress	Patient Health Questionnaire-9 item (PHQ-9)	Difference in change scores	Mean score	T1–T2; T1–T3; T1–T4
Psychological distress	Generalized Anxiety Disorder 7-item (GAD-7)	Difference in change scores	Mean score	T1–T2; T1–T3; T1–T4

^a^A patient-only measure

#### Patient sexual function (primary outcome measure)

Sexual function is assessed using the Female Sexual Function Index (FSFI [67]), a widely used sexual function measure with established validity in breast cancer [68, 69]. The FSFI is a 19-item multidimensional measure of sexual function in assessing various aspects of sexual function including desire, arousal, lubrication, orgasm, pain during sex, and sexual satisfaction. The total score will be used, as it reflects women’s overall sexual function and because total FSFI scores are sensitive to increases in similar interventions [42, 43].

#### Secondary outcome measures

##### Partner sexual function

Partner sexual function is assessed using the International Index of Erectile Function (IIEF) [70] if the partner is male or the FSFI [67], described previously, if the partner is female. The IIEF is a 15-item multidimensional measure of sexual function that assesses various aspects of male sexual function including desire, erectile function, orgasm, and sexual satisfaction. The IIEF is the most widely used measure to assess male sexual function and has been used successfully in a multitude of healthy and medical populations [71].

##### Patient sexual distress

Patient sexual distress is assessed using the Female Sexual Distress Scale-Revised (FSDS-R [72]). The FSDS-R is a 13-item validated measure of female sexual distress designed for use in women’s sexual medicine trials [72] that measures the degree of distress and dissatisfaction related to a woman’s sex life over the past 30 days. The FSDS-R has been used in both observational and intervention studies with breast cancer survivors [5, 25, 73].

##### Patient and partner relationship intimacy

Patient and partner relationship intimacy is assessed using the Miller Social Intimacy Scale (MSIS [74]). The MSIS is a 17-item scale assessing emotional intimacy, closeness, and trust toward an individual’s partner that has been used in trials assessing psychosocial and sexual couple-based interventions of psychosocial interventions for patients with cancer [54, 75, 76].

Patient and partner relationship quality is assessed using the 7-item Dyadic Adjustment Scale (DAS-7 [77, 78]). The DAS-7 provides information on relationship quality comparable to that obtained when using the full 32-item measure and can distinguish between couples who are distressed versus well-adjusted on the full scale [78]. The DAS-7 has been used successfully in studies in breast cancer [79].

##### Patient and partner psychological distress

Patient and partner psychological distress are assessed using the Patient Health Questionnaire-9 item (PHQ-9 [80]) and Generalized Anxiety Disorder 7-item (GAD-7 [81]), both of which are validated and commonly used measures to assess depression and anxiety, respectively, in cancer and other medical populations [82].

### Intervention mediators

#### Patient self-efficacy for coping with sexual concerns

Patient self-efficacy with sexual concerns is assessed through three items measuring patients’ confidence in their ability to communicate effectively about sexual concerns, deal effectively with sexual concerns, and enjoy sexual intimacy despite physical limitations. These items were developed using standard methods for constructing self-efficacy scales according to social cognitive theory [83] and have shown excellent psychometric properties when used as a single scale as well as sensitivity to the IE intervention [55].

#### Patient sexual communication

Patient sexual communication is assessed using 6 items from the Dyadic Sexual Communication Scale (DSCS [84]), which assesses the perceived quality of communication about sex in the context of the intimate relationships and has been used successfully in cancer studies [53, 85]. Previously, we found that these 6 items (items 2, 3, 4, 10, 11, and 12) had 95% correlation with the 13-item scale in a sample of breast cancer survivors (Reese JB & Handorf E: Establishing the reliability of an abbreviated 6-item dyadic sexual communication scale for use with breast cancer survivors, unpublished).

#### Intervention moderators

The potential moderators of patient age (< 45 versus > 45) at diagnosis and race/ethnicity (White versus non-White) will be assessed using medical records and patient self-report, respectively. Age is selected as a moderator for the same reason as it is selected as a stratifier variable, in that younger and older women may differ in their sexual function [9, 56] and could thus differ in their response to the intervention. Race/ethnicity is included as a potential moderator because there is little data on whether couple-based sexuality interventions are equally effective in breast cancer survivors from different racial/ethnic backgrounds.

#### Other measures

Socio-demographic characteristics such as education, sexual orientation, marital and work status, race/ethnicity, income, and relationship length are assessed through self-report. Clinical patient characteristics including menopausal status and types and dates of treatments are obtained through medical chart review. Medical comorbidity data for patients and partners are obtained by a validated self-report comorbidity measure (Self-Administered Comorbidity Questionnaire; SCQ) (Sangha O, Stucki G, Liang MH, Fossel AH, Katz JN: The Self-Administered Comorbidity Questionnaire: a new method to assess comorbidity for clinical and health services research, unpublished). In addition, a brief measure is used to assess intervention credibility [86] in both conditions (i.e., logic, helpfulness, and competence of the counselor) after Session 1 and at the post-intervention time point (T2).

### Study arms

#### Intervention overview

Both intervention conditions are administered over the telephone to both members of the couple jointly and are designed to be of equal duration and to contain material of interest to both patients and partners. Both conditions are delivered according to a standard intervention protocol, consisting of an interventionist manual and corresponding participant handouts. All session calls are audio recorded. The first session for both interventions is designed to last approximately 75 min, and the remainder to last 60 min each.

#### Interventionists

Interventions are conducted by interventionists with a master’s or doctoral degree in a mental health field certified competent in delivery of the interventions by the PI. All interventionists complete an in-depth training protocol consisting of background readings on key topics including common sexual and non-sexual side effects of breast cancer treatment, sexual response [87], key techniques such as cognitive behavioral couple therapy [49] and sensate focus therapy [88], theoretical models [45, 89, 90], and cultural considerations [91, 92], and the intervention protocols (manuals, participant handouts); a training workshop which includes review of readings, protocols, and specific skills, role plays, and discussions; listening to and discussing full cases of each condition; and finally, completing full test cases of each condition and corresponding supervision. A key element of training is ensuring that the interventionists are able to maintain fidelity to each of the two intervention manuals while establishing rapport and therapeutic alliance. To this end, the interventionists complete session adherence forms after each session in order to capture information on their perceived completion of the intervention session components, the case conceptualization, and to identify difficulties they had in delivering the interventions as intended. These processes help catch possible “drift” in the delivery of intervention material over time or across conditions. Supervision with the PI (Reese) and Duke site PI (Porter) occurs regularly and includes review of session audio recordings and supervisor-completed adherence and performance forms, as well as discussions of related intervention delivery and case issues. A random selection of at least 10% of sessions will be reviewed to assess intervention fidelity by an independent reviewer not involved with the intervention delivery.

#### Intimacy Enhancement (IE)

The Intimacy Enhancement (IE) intervention incorporates education and training in skills for coping with sexual concerns. The IE intervention is designed to address women’s sexual concerns that are physical (e.g., vaginal dryness), emotional (e.g., loss of libido), or interpersonal in nature (e.g., changes in sexual scripts due to breast changes) [93]. Content also integrates a framework for coping with sexual concerns that emphasizes flexibility in thoughts and behaviors, and encourages more inclusive thinking about how sex and intimacy are enacted within the relationship [89, 90]. The four sessions and their content and structure are described in Table 3. Participant handouts reinforce educational material, provide opportunities for interactive exercises, and reinforce skills learned during the sessions. Weekly home behavioral skills practice are reviewed at the beginning of each session, including proceeding through a stepped set of sensate focus exercises (i.e., non-demand sensual touching) [51], with the goal of reducing avoidance of physical intimacy and increasing comfort with sexual activity.

**Table 3:** Intimacy Enhancement intervention session overview

Session (length)	Title	Content	Home practice
1 (75 min)	Introduction to Intimacy Enhancement	1. Sexual response cycle 2. Breast cancer effects on sex and intimacy 3. Intimacy challenges and goal-setting 4. Introduction to physical intimacy skills (sensate focus) 5. Treatments and sexual aids	1. Read educational handouts 2. Sensate focus Practice 1
2 (60 min)	Enhancing Intimacy Through Communication	1. Effective communication about sex and intimacy 2. Challenges in communicating effectively about sex and intimacy 3. Communication skills practice	1. Communication skills practice 2. Sensate focus Practice 2
3 (60 min)	Enhancing Intimacy Through Thinking and Doing	1. Positive and negative thought cycles 2. Flexible and inflexible thinking about sex and intimacy 3. Identifying and changing negative/inflexible thoughts 4. Broadening range of intimate activities (intimacy-building activities)	1. Intimacy-building activities 2. Sensate focus Practice 3
4 (60 min)	Planning Ahead for Intimacy	1. Review of skills and education 2. Evaluate progress toward goals 3. Plan for continued skills practice 4. Anticipate and plan for challenges (“red flags” for maintaining intimacy)	

#### Living Healthy Together (LHT)

The LHT intervention focuses on delivering education and support across a range of topics relevant to women with breast cancer. Research has found that health-related concerns including social support, sleep and fatigue, stress, and diet are among the top concerns for breast cancer survivors [94, 95], and thus these topics were selected for inclusion. To increase participant engagement, the material in this condition includes participant self-assessment related to health habits and discussion of challenges in achieving or sustaining beneficial health behaviors during the sessions. The LHT intervention demonstrated excellent credibility and acceptability in a pilot study [55]. This condition includes education on breast cancer experience and finding support (Session 1), stress and stress management (Session 2), fatigue and sleep (Session 3), and nutrition and physical activity (Session 4). Couples in this condition are encouraged to engage with the material between sessions by completing readings, trying out strategies, and seeking out resources and information*.*

### Statistical plan

#### Overview of statistical analyses

First, descriptive statistics will be used to characterize sample covariates. Outcome variables will be assessed for normality and, if necessary, normalizing transformations may be applied. We will determine whether the enrolled sample differs from eligible study refusers on key socio-demographic and clinical factors obtained during screening (e.g., age, treatments, severity of sexual concerns) using two-sample *t* tests or chi-square tests. Study completers will also be compared to non-completers on these variables, and logistic regression will be used to understand what factors are predictive of drop-out. Variables showing significant imbalances will be included in subsequent models as covariates. We will account for missing data using multiple imputation by chained equations (MICE) [96–98], which provides valid inferences provided that the probability of having missing data (i.e., drop-out) only depends on the observed data (e.g., treatment arm, baseline sexual function). For primary and secondary outcome analyses, differences in mean change scores for the outcomes will be used (see Table 2 for outcome definitions). No interim analyses are planned.

#### Primary aim

The primary analysis will examine whether, relative to the LHT intervention, the IE intervention leads to greater increases in patient sexual function at all three post-treatment assessments in a mixed-effects regression model. Pre-treatment sexual function scores and time (categorical) will be included as covariates. Intervention by time interactions will test the intervention effect at each follow-up time. Subject-specific random intercepts will account for within-subject variability. Intervention effects on patient sexual function at each follow-up are tested using *F* tests of combined main and interaction effects.

#### Secondary aims

Analyses of intervention effects on partner sexual function and patient sexual distress will use similar mixed-effects models as those described under the primary aim. Partner sexual function will generally be measured and analyzed using a male-specific scale. In additional analyses, potential same-sex partners’ sexual function data will be handled by categorizing all scores as dysfunctional versus functional (using clinical cut-offs), and estimating the intervention effect via logistic mixed-effects regression models [99, 100], controlling for partner sex. Relationship intimacy and quality and psychological distress will be assessed using identical measures in both patients and partners. Multilevel modeling (MLM) [101] will be used to test for differences in these outcomes between the IE and LHT groups over time. These models include all main effects and interactions among time (categorical), treatment, and role (patient/partner). Models also include random intercepts for patients and partners, as well as the correlation between the intercepts (i.e., if a patient is high in average distress across time, is the partner also high in distress?). Models will include a time-specific correlation between the partners’ residuals (i.e., if a patient is distressed at a particular time point, is the partner also distressed at that time?).

#### Mediators and moderators

We will evaluate whether pre-intervention to post-intervention changes in either sexual communication and/or in self-efficacy for coping with sexual concerns mediate treatment effects on sexual function using the causal inference framework for mediation described by VanderWeele [102, 103]. Initial models will assess mediation at 3 months follow-up; if evidence of mediation is found, 6 months follow-up will be explored in separate models. Effects will be estimated via structural equation modeling, and covariates (age, race, etc.) will be included to account for possible mediator/outcome confounding. Separate models will be fit for the two proposed mediators.

We will explore whether both age (< 45 versus > 45) and race/ethnicity (White versus non-White) moderate the intervention effects. In separate models, we will add each moderator and interactions between the moderator, intervention, and time point to the model of Aim 1. A significant three-way interaction will provide evidence that the treatment effect differs by age or race. However, as this study is not powered to detect interactions, we will consider interaction effects with a magnitude > (0.5 * main effect) to be of interest.

### Sample size estimate/power calculations

Power calculations are based on Aim 1. For patient sexual function, a change of 5 points would be clinically meaningful (scale range 2–36, standard deviation [SD] ≈ 10). Based on prior data, we anticipate that the SD of the change scores will be 8.2, and that there will be an attrition rate of 24%, resulting in 92 analyzable couples at 6 months follow-up. We will therefore have 82% power to detect a difference of 5 points in change scores, assuming a two-sided test with 5% type I error.

### Ethical aspects

The trial has been approved by the Institutional Review Boards at Fox Chase Cancer Center (Protocol 18-1025) and Duke University Medical Center (Protocol Pro00100404). All study faculty and staff have been trained in principles of ethical conduct of human subjects’ research and in compliance with study procedures. Study participants are informed that their participation or decision not to participate or to withdraw will not have an effect on their receipt of healthcare at their respective institutions. The financial incentives for participation are consistent with those offered in comparable couple-based studies and are meant to ethically compensate participants for completion of repeated study surveys. Given the minimal risk of this study, a Data and Safety Monitoring Board was deemed unnecessary. The institutional research review committees at the respective study sites review all study activities annually including ethical conduct, regulatory compliance, and recruitment and retention, and can initiate an independent study audit at any time. The study sponsor also monitors study progress annually including sufficient recruitment and retention. In addition, the investigator team meets at least monthly to discuss enrollment targets and treatment fidelity. The study coordinator is responsible for ensuring that data is complete from both sites and for tracking enrollment at both sites. Adverse events across conditions will be reviewed annually; issues with intervention delivery and other unintended consequences of either intervention are discussed during regular supervision meetings. Significant protocol modifications will be approved by relevant Institutional Review Boards and will be reported to relevant parties (e.g., ClinicalTrials.gov) in a timely fashion. Couples have the option of discontinuing their participation in the study at any time, and this is indicated in the consent forms. Adverse events will be reported immediately to the PI, tracked, and responded to according to regulatory guidelines. In the rare case of distress (individual or relational) so severe that continuation of the intervention sessions or of the study procedures is judged to interfere with the participant’s well-being or timely receipt of necessary care, the couple will be counseled to discontinue and will be referred for appropriate care. There are no criteria for the modification of the study interventions. As harm from this type of study is rare, there are no provisions in place for ancillary, post-trial, or compensation for study-related harms.

## Discussion

Upon completion of this study, we will have provided a rigorous test of the efficacy of an innovative intervention addressing sexual concerns and enhancing the sexual, relationship, and psychosocial outcomes for breast cancer survivors and their partners. This intervention is unique from other couple-based interventions in cancer by focusing explicitly on the intimate relationship and by integrating the partner fully into the activities aimed at building couples’ skills for coping with sexual concerns and enhancing intimacy. Results of this study will also provide information on theoretically based mediators of the IE intervention effects, and potential moderators of treatment efficacy.

The IE intervention has several important features that may enhance its impact, including a strong theoretical basis in social cognitive theory, incorporation of evidence-based skills practices from couple therapy and sex therapy, grounding in formative qualitative research with breast cancer survivors, and the use of a telephone format, which has several advantages over face-to-face and web-based options. Specifically, a telephone format may decrease barriers to access for couples who cannot make use of a comparable web-based intervention due to lack of Internet or computer access or literacy, and it makes it possible for couples to participate who would be unable to attend in-person visits due to geographical limitations, cost, or the burden of travel. This format may be particularly well-suited to the needs of post-treatment breast cancer survivors, who report interest in obtaining help for sexual concerns [104] but make fewer in-person visits, and may be preferred over face-to-face interventions for discussing sexuality [53].

This study has several strengths including the use of an active control condition that equates for interventionist time and attention, examination of a range of important patient and partner sexual, relationship, and psychological outcomes, and examination of treatment mediators and moderators. Understanding treatment mediators is important given that there is a paucity of research on mechanisms underlying the efficacy of sexual function interventions in cancer [47, 105], while exploring intervention moderators could help us determine whether to adapt the IE intervention to address the needs of certain breast cancer patient subgroups.

This study also has several limitations that should be considered. For instance, because both intervention conditions are designed for couples, only breast cancer survivors who are partnered may participate in the trial, thus excluding unpartnered survivors who have sexual concerns. In addition, although the trial is open to both opposite-sex and same-sex couples, given our past experience, we anticipate that most couples will likely be heterosexual. Thus, the study will not be powered to determine whether effects of the IE intervention could differ for patients by sexual orientation, although examining this would be an important step in advancing the research on evidence-based sexual function interventions for sexual and gender minority cancer survivors. Despite these limitations, this study represents a critical piece of a larger program of research that aims to advance clinical care through the development, evaluation, and dissemination of evidence-based interventions to improve the sexual health and QOL of those affected by cancer. The findings of this trial will be disseminated to researchers and the public through the study’s entry on ClinicalTrials.gov, through publication in peer-reviewed journals, and through presentation of the findings to the scientific community at scientific conferences.

In conclusion, sexual concerns for breast cancer survivors frequently go unaddressed and evidence-based interventions are needed, particularly those that integrate survivors’ partners. With the proposed study completed, we will be well-equipped to determine critical next steps in this program of research. For instance, if we find that the IE intervention is effective, we may consider expanding it for use with other cancer populations in need of evidence-based interventions addressing sexual concerns (e.g., head and neck cancer, patients with advanced cancer). We may also consider a pragmatic trial that would allow us to determine whether delivering this intervention in the “real world” could sustain effects. Depending on the findings from the mediation analyses, another interesting next step could be to examine whether individual component(s) of the IE intervention (e.g., communication skills training; sensate focus practice) would hold up against the full intervention in a controlled trial. Finally, findings of the moderator analysis could help us determine whether to consider adapting the IE intervention to address the needs of particular subgroups of patients with breast cancer.

### Trial status

Both sites are actively recruiting participants for this trial. Recruitment for the trial began in May 2019 and is expected to continue until November 2022. This manuscript describes Version 6 of the study protocol, dated 07/02/2019.

## Supplementary information

**Additional file 1.** Detailed SPIRIT checklist of items for the study.

## Data Availability

Study data and relevant materials from the trial described in this manuscript will be retained and archived by the primary study site for a minimum of 3 years after study completion as per National Institutes of Health (NIH) policy on record retention. There are no plans for publicly sharing the trial data. No materials (biological specimens) are collected as part of this trial.

## Abbreviations

DAS-7: Dyadic Adjustment Scale-7 item
DSCS: Dyadic Sexual Communication Scale
DUMC: Duke University Medical Center
ECOG: Eastern Cooperative Oncology Group
FCCC: Fox Chase Cancer Center
FSDS-R: Female Sexual Distress Scale-Revised
FSFI: Female Sexual Function Index
GAD-7: Generalized Anxiety Disorder 7-item
IE: Intimacy Enhancement
IIEF: International Index of Erectile Function
LHT: Living Healthy Together
MLM: Multilevel modeling
MSIS: Miller Social Intimacy Scale
PCM: Patient Care Monitor
PHQ-9: Patient Health Questionnaire-9 item
PI: Principal investigator
QOL: Quality of life
SCQ: Self-Administered Comorbidity Questionnaire
SD: Standard deviation
